# Monogenic forms of systemic lupus erythematosus: new insights into SLE pathogenesis

**DOI:** 10.1186/1546-0096-10-21

**Published:** 2012-08-10

**Authors:** Alexandre Belot, Rolando Cimaz

**Affiliations:** 1 Pediatric nephrology and rheumatology Unit, Hôpital Femme Mère Enfant, Lyon, Bron, Université de Lyon, Lyon, CNRS UMR5239, France; 2 Rheumatology Unit, Anna Meyer Children's Hospital, University of Florence, Florence, Italy

**Keywords:** SLE genetics, Mendelian, Complement deficiency, Interferon-alpha, Pediatrics

## Abstract

The pathogenesis of Systemic Lupus Erythematosus (SLE) is complex and remains poorly understood. Infectious triggers, genetic background, immunological abnormalities and environmental factors are all supposed to interact for the disease development. Familial SLE as well as early-onset juvenile SLE studies make it possible to identify monogenic causes of SLE. Identification of these rare inherited conditions is of great interest to understand both SLE pathogenesis and molecular human tolerance mechanisms. Complement deficiencies, genetic overproduction of interferon-α and apoptosis defects are the main situations that can lead to monogenic SLE.

Here, we review the different genes involved in monogenic SLE and highlight their importance in SLE pathogenesis.

## 

Systemic lupus erythematosus (SLE) is a complex disease: environmental factors (e.g. infections), immunological defects (responsible for tolerance breakdown), and genetic factors (mostly thought to be polygenic and associated to genetic polymorphism [1,2]) all play a role in its development. Rarely, lupus can be secondary to single gene mutations; we summarize recently discovered monogenic forms of lupus, and highlight the impact of these gene mutations on SLE pathogenesis.

## Review

### Complement defects: apoptotic cell and immune complexes clearance deficiency

Primary complement defects, especially in early components of the classical pathway, can lead to an increased susceptibility to SLE [3]. However, less than 1% of SLE cases are associated with complement deficiencies, and conversely complement deficiencies are not always associated to SLE [4]. C1q, C1s and C1r complete deficiencies are rare and associated with a high risk to develop pediatric SLE (estimated to 93% for C1q and 66% for C1s/r). C1q deficiency is associated with cutaneous rash in 90% of case and glomerulonopehritis in around 1/3 of cases [5]. Notably, the incidence of anti–double-stranded DNA antibodies "(anti-dsDNA)"is low. C4 deficiency is also strongly associated with SLE development (around 75%) [6-8] (Table 1). Homozygous C2 deficiency, which is the most frequent hereditary deficiency in classical pathway complement components (1/10,000 to 1/30,000 among caucasians), is associated with SLE in only 10 to 30% of the cases, suggesting that exogenous factors may be also involved. In all cases, early-onset disease and association with recurrent pyogenic or neisserial infections should evoke the diagnosis.

**Table 1:** Complement deficiencies

**Complement deficiency**	**Locus**	**Inheritance pattern**	**Clinical manifestations**	**Infection susceptibility**
C1q	1p36.3-p34.1	AR	Nephritis, CNS involvement, photosensitivity	Encapsulated bacteria
C1r/C1s	12p13	AR	Nephritis	Encapsulated bacteria
C4	6p21.3	AR	Multiorgan involvement; glomerulonephritis	Encapsulated bacteria
C2	6p21.3	AR	Photosensitivity and articular involvement; mild or absent renal, neurological or pleuropericardial involvement	Pyogenic infections; encapsulated bacteria; Streptococcus pneumoniae sepsis and meningitis
C3	19q13	AR	Malar rash, photosensitivity, arthralgia and Raynaud’s phenomenon	Recurrent pyogenic infections
C5-C9: MAC	C5/9p34.1, C6-C7/5p13, C8A-C8B/1p32, C8G/9, C9/5p13	AR	Multiorgan involvement	Neisserial infections

**Figure 1:**
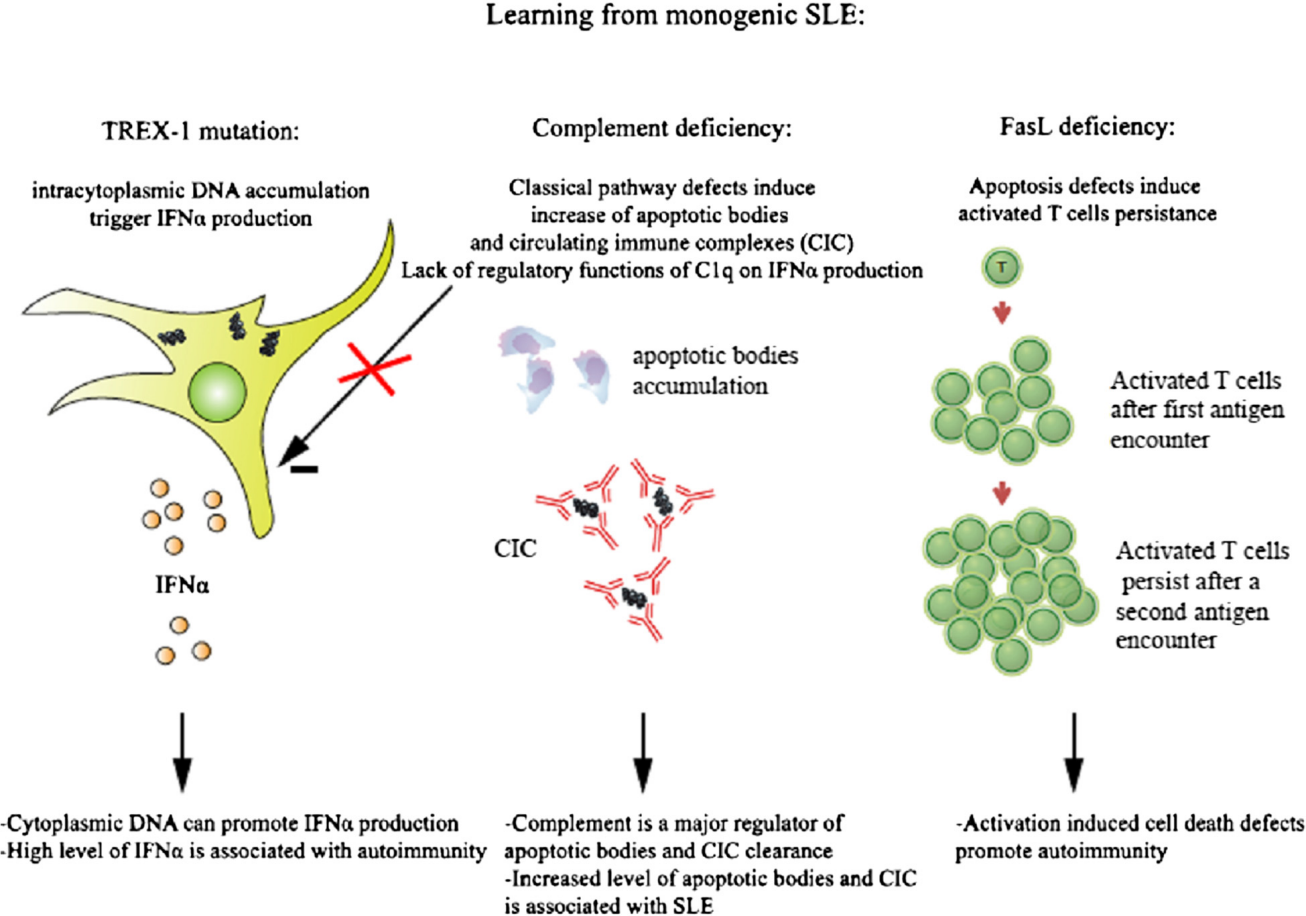
Schematic views of monogenic SLE pathogenesis.

Complement deficiencies demonstrate the crucial role of complement in maintenance of tolerance (Figure 1). Early components of the classical pathway (especially C1q) help clearing apoptotic cells, thus decreasing the number of autoantigens [9]. Another role of complement is to process antibodies and eliminate circulating immune complexes, thus decreasing or avoiding vascular deposition. Interestingly, complement plays a role in T and B cell activation as well, and complement deficiency may upset the balance of lymphoid cell activation[10]. Finally, C1q has also been shown to inhibit *in vitro* interferon IFN-α production by plasmacytoid dendritic cells [11], and its deficiency can lead to defective suppression of IFN-α in response to immune complex-containing nucleoproteins [12].

### Apoptosis defects

Apoptosis defects are thought to be involved in SLE pathogenesis as autoreactive B and/or T cells might survive death signals when they are ongoing central or peripheral tolerance. Nevertheless, to date there is no evidence that apoptosis-related gene defects involving B or T cell tolerance are directly involved in the pathogenesis of SLE. Autoimmune lymphoproliferative syndrome (ALPS) is the human counterpart of the *lpr* mouse, a widely explored model of murine lupus. Molecular mechanisms of ALPS are related to deficiencies of T cell apoptosis. Mutations in the Fas/FasL pathway underlie ALPS, which is characterized by lymphoproliferation in lymphoid organs associated with multiple autoimmunity [13]. One single case has been reported with classical features of SLE [14].

### Interferon (IFN)-α hyperproduction

Genomic approaches have shown that human SLE leukocytes homogeneously express type I interferon (IFN)-induced transcripts [15]. In addition, large-scale genetic analyses have demonstrated that genes involved in IFN-α pathway such as *IRF5IRF7* both expressed downstream of the endosomal TLR or *STAT-4*, transcriptional factor induced by Interferon type I were associated with SLE [16]. In the last years, studies on early-onset SLE and familial SLE led to the identification of new genes involved in IFN-α production (Table 2). This all started with the identification of the genes responsible for Aicardi-Goutieres syndrome (AGS) [17]. AGS is a rare genetic disorder occurring within the first few weeks of life that can mimic maternofetal infections, with an inflammatory encephalopathy. This autosomal recessive disease is associated with high IFN-α production [18]. Some children with AGS develop an early-onset form of SLE [19,20].

**Table 2:** Main features of mendelian SLE

**Gene mutated/protein**	**Chromosome**	**Inheritance**	**Clinical features**
*TREX1*/TREX1	3p21	AD	Chilblain lupus, intracerebral calcifications
*DNAse I/*DNase I	16p13	AD	Systemic lupus, Sjögren syndrome,high levels of antinucleosomal antibodies
*DNAse IL3/*DNase1L3	3p14	AR	Early-onset SLE, antinuclear antibodies, anti-dsDNA, ANCA
*AGS5*/SAMHD1	20q11	AD	Chilblain lupus, intracerebral calcifications, mental retardation
*ACP5*/TRAP	19p13	AR	Growth retardation, spondyloenchondrodysplasia, SLE, Sjögren, vitiligo, myositis, Raynaud, ANA, anti-dsDNA

AD = autosomal dominant; AR = autosomal recessive.

Nucleic acid are able to initiate an immune response, activating membrane receptors such as Toll-like receptors (TLR) or cytosolic sensors, involved in IFN-α production [21]. Defective clearance of self-derived nucleic acids can cause severe IFN-associated autoimmunity (Figure 1). One major mechanism by which these extracellular nucleic acids cause autoimmunity is through activation of TLR7 and TLR9 on autoreactive B cells [22,23]. Recently, it has been shown that TREX1 deficiency results in endogenous DNA accumulation and IFN-α production, independently from TLRs [24].

#### Rare cases of nuclease defects are responsible for monogenic lupus

DNAse type III, also called TREX1, is the main 3’-5’DNA exonuclease and has been shown to down-regulate IFN-stimulatory DNA response [24]. TREX1 knockout mouse develop an inflammatory cardiopathy [25]. Interestingly, TREX1 deficiency is responsible for intracellular DNA accumulation and TLR independent- IFN-α production[24]. *TREX1* mutations in humans lead to Aicardi-Goutieres syndrome, chilblain lupus and represent the more common cause of monogenic lupus. Indeed, systematic *TREX1* mutation screening in adult lupus patients revealed 0.5 to 2% heterozygosity, thus making *TREX1* mutations the most frequent form of monogenic lupus [26,27].

#### Other gene involved in Aicardi Goutières syndrome

SAMHD1 is a protein, encoded by *AGS5*, that is upregulated in response to viral infections and may have a regulator role on immune system and cerebral vascular homeostasis [28,29]. One case of chillblain lupus in a 3-year-old boy with epilepsy was first diagnosed with a mutation in *AGS5* gene [30]. Two additional cases have been reported with typical chillblain lupus, without central nervous system involvement [31]. *AGS5* mutations can be associated with arthritis, chronic ulcers, mental retardation and microcephaly. Plasmatic IFN-α is increased, even though SAMHD1 deficiency has not yet been directly linked to IFN overproduction.

#### Spondyloenchondrodysplasia (SPENCD)

*ACP5* is another gene, encoding tartrate-resistant acid phosphatase (TRAP), that has been associated to an immuno-osseous disease: the spondyloenchondrodysplasia (SPENCD). This syndrome is associated with platispondily, growth retardation and enchondromatosis. Various immunological findings have been reported including typical SLE with malar rash, lupus nephritis, antiphospholipid syndrome and anti-dsDNA [32]. Osseous anomalies can be subtle. TRAP is expressed in bone and in immune cells (mainly osteoclasts and dendritic cells) and is involved in bone resorption, even though its precise physiological role remains to be defined. Osteopontin (OPN, a substrate of TRAP) is a bone matrix protein involved in osteoclast adhesion and migration, and is dephosphorylated by TRAP [33,34]. In TRAP-deficient mice, OPN accumulates both around osteoclasts and in intracellular vacuoles, suggesting that TRAP is required for processing and/or degradation of OPN [16]. Interestingly, OPN accumulates in serum, urine and cells cultured from TRAP-deficient individuals and patients’ dendritic cells exhibit an altered cytokine profile, and are more potent than control cells in stimulating allogeneic T cell proliferation in mixed lymphocyte reactions [35]. OPN and IFN-α levels are both elevated in lupus patient’s sera and seems correlated [36]. In mouse, OPN is essential for IFN-α production, downstream of the Toll-like-receptor 9 in plasmacytoïd dendritic cells [37]. Taken together, TRAP deficiency may drive an inflammatory T cell response and promote IFN-α production in human.

#### DNASE1/DNASE1L3 mutation

DNAse type I is a widespread endonuclease that can be found in blood and urine. DNase1 deficiency in mouse induces the presence of ANA and the deposition of immune complexes in glomeruli [38]. Two unrelated cases of juvenile SLE were reported with a mutation in DNAse type 1 [39], and exhibited very high levels of antinucleosomal antibodies. This variant represent a very rare cause of lupus and further exploration in a large UK cohort of 170 SLE patients did not found any mutation [40]. Recently, a group from Saudi Arabia has identified *DNASE1L3* as a new gene mutated in familial cases of juvenile SLE [41]. Mutated patients presented with association of anti-nuclear antibody, anti-dsDNA and ANCA. The link to SLE pathogenesis is unknown, but may be (as in *TREX1* mutations) related to DNA accumulation, thus triggering IFN-α production.

### Chronic granulomatous disease (CGD)

CGD is characterized by recurrent life-threatening infections by bacteria and fungi, due to severely impaired phagocyte intracellular destruction. CGD is caused by defects of NADPH (nicotinamide adenine dinucleotide phosphate) oxidase system, which is responsible for the generation of superoxide and other reactive oxygen species in phagocytic cells. The X-linked form, caused by mutations of the *CYBB* gene, accounts for more than 75% of the cases [42]. In the large U.S. series of 368 CGD patients, ten (2.7%) affected patients presented concomitantly discoid lupus (DLE) and 2 (0.5%) SLE. A large number of first-degree female relatives were also reported as having SLE or discoid lupus, while infection susceptibility was not increased. Cale et al. [43] investigated 19 mothers who carried the X-linked CGD allele for the presence of lupus manifestations. Remarkably, 12 of them presented photosensitivity, seven arthralgia, and eight had mouth ulcers. Anti-nuclear antibodies were positive in five, one had anti-dsDNA and another a lupus anticoagulant. The link between CYBB mutation and lupus may arise from the apoptosis defect of neutrophils in CGD patients, characterized by an impaired exposure of phosphatidyl serine on neutrophil membrane [44]. Moreover, in CGD neutrophil apoptosis is associated to diminished production of anti-inflammatory mediators [5]. Of note, neutrophils are now have been shown to be important in lupus pathogenesis, especially since mature SLE neutrophils are primed in vivo by type I IFN and die upon exposure to SLE-derived anti-ribonucleoprotein antibodies, releasing neutrophil extracellular traps (NETs) which contain DNA as well as large amounts of proteins that facilitate the uptake and recognition of mammalian DNA by plasmacytoid dendritic cells [45]. Indeed, SLE NETs activate pDCs to produce high levels of IFN-α in a DNA- and Toll-like receptor 9-dependent manner.

Altogether, these data might suggest that CYBB and other CGD-related genes could be lupus-susceptibility genes.

## Conclusions

Early onset lupus, familial lupus and syndromic lupus are rare situations that can lead to the identification of a unique gene responsible for the disease. Identification of this monogenic susceptibility to lupus can help to understand both lupus pathogenesis and tolerance breakdown in human immunology. To date, complement system deficiency, apoptosis defects and interferon overproduction have been confirmed as responsible for susceptibility to lupus. New genetic techniques such as exome sequencing could help to discover new genes and give insights in understanding SLE pathogenesis as well as molecular mechanisms of tolerance maintenance.

## Abbreviations

AGS: Aicardi-Goutieres syndrome; CGD: Chronic granulomatous disease; IFN: Interferon; NETs: Neutrophil extracellular traps; SLE: Systemic lupus erythematosus; SPENCD: Spondyloenchondrodysplasia; TLR: Toll-like receptor.

## Competing interests

The authors declare that they have no competing interest.

## Authors’ contributions

AB and RC elaborated the draft and wrote the paper. Both authors read and approved the final manuscript.
